# SARS-CoV-2 Infection: New Molecular, Phylogenetic, and Pathogenetic Insights. Efficacy of Current Vaccines and the Potential Risk of Variants

**DOI:** 10.3390/v13091687

**Published:** 2021-08-25

**Authors:** John Charles Rotondo, Fernanda Martini, Martina Maritati, Chiara Mazziotta, Giulia Di Mauro, Carmen Lanzillotti, Nicole Barp, Altea Gallerani, Mauro Tognon, Carlo Contini

**Affiliations:** 1 Department of Medical Sciences, University of Ferrara, 44121 Ferrara, Italy; fernanda.martini@unife.it (F.M.); martina.maritati@unife.it (M.M.); mzzchr@unife.it (C.M.); giulia.dimauro@unife.it (G.D.M.); lnzcmn@unife.it (C.L.); nicole.barp@edu.unife.it (N.B.); altea.gallerani@edu.unife.it (A.G.); mauro.tognon@unife.it (M.T.); 2 Center for Studies on Gender Medicine, Department of Medical Sciences, University of Ferrara, 64/b, Fossato di Mortara Street, 44121 Ferrara, Italy; 3 Laboratory for Technologies of Advanced Therapies (LTTA), University of Ferrara, 44121 Ferrara, Italy

**Keywords:** Severe acute respiratory syndrome coronavirus 2, SARS-CoV-2, CoV, coronaviruses, coronavirus disease 2019, COVID-19, spike protein, variants, vaccine, zoonosis

## Abstract

Severe acute respiratory syndrome coronavirus 2 (SARS-CoV-2) is a newly discovered coronavirus responsible for the coronavirus disease 2019 (COVID-19) pandemic. COVID-19 has rapidly become a public health emergency of international concern. Although remarkable scientific achievements have been reached since the beginning of the pandemic, the knowledge behind this novel coronavirus, in terms of molecular and pathogenic characteristics and zoonotic potential, is still relatively limited. Today, there is a vaccine, or rather several vaccines, which, for the first time in the history of highly contagious infectious diseases that have plagued mankind, has been manufactured in just one year. Currently, four vaccines are licensed by regulatory agencies, and they use RNA or viral vector technologies. The positive effects of the vaccination campaign are being felt in many parts of the world, but the disappearance of this new infection is still far from being a reality, as it is also threatened by the presence of novel SARS-CoV-2 variants that could undermine the effectiveness of the vaccine, hampering the immunization control efforts. Indeed, the current findings indicate that SARS-CoV-2 is adapting to transmission in humans more efficiently, while further divergence from the initial archetype should be considered. In this review, we aimed to provide a collection of the current knowledge regarding the molecular, phylogenetic, and pathogenetic insights into SARS-CoV-2. The most recent findings obtained with respect to the impact of novel emerging SARS-CoV-2 variants as well as the development and implementation of vaccines are highlighted.

## 1. Introduction

Coronaviruses (CoVs) are enveloped RNA viruses belonging to the *Coronaviridae* family [1,2,3]. These viruses are classified into four major genera known as alpha-, beta-, gamma-, and delta-CoV [4,5]. CoVs can infect both animals and humans [3,6,7]. Phylogenetic analyses revealed that alpha- and beta-CoVs mostly infect bats and rodents, while gamma- and delta-CoVs infect birds [8]. A key characteristic of CoVs’ infective potential is that they can adapt to different hosts from a variety of ecological niches, as their mutation rates are high. Indeed, in addition to birds and rodents, CoVs infect a plethora of species, such as rabbits, cats, pigs, dogs, ferrets, and horses, whereas some of them also infect humans [6,9]. 

Since the beginning of the 21st century, three different CoVs caused major outbreaks of fatal pneumonia in humans by infecting the respiratory tract. Severe acute respiratory syndrome coronavirus (SARS-CoV-1) has been found to be the first CoV outbreak, which started in 2002 at Foshan, China. Ten years later, Middle East respiratory syndrome coronavirus (MERS-CoV) caused the second outbreak and originated in Jeddah, Saudi Arabia. Presumably, in December 2019 at Wuhan, China, SARS-CoV-2, the novel homologous strain of SARS-CoV-1, caused the major outbreak of the CoV pandemic named coronavirus disease 2019 (COVID-19) [10,11,12]. SARS-CoV-2 is the seventh CoV known to infect humans [13]. Indeed, four other human coronaviruses (HCoVs) have been identified, named HCoV-229E, HCoV-OC43, HCoV-NL63, and HCoV-HKU1 [2]. All these viruses passed from animals to humans in different ways: (i) HCoV-229E passed from bats to humans though alpacas; (ii) HCoV-OC43 originated in rodents and transmitted to humans though cattle; and (iii) SARS-CoV-1 and MERS-CoV originated in bats and infected humans from carnivores and dromedary camels, respectively [7]. In humans, CoVs usually infect the respiratory tract, thereby inducing mild respiratory symptoms, such as cold and diarrhea in immunocompetent patients. Since infecting the respiratory tract, SARS-CoV-1, MERS-CoV, and SARS-CoV-2 exhibit higher pathogenicity than other HCOVs, causing severe pneumonia with a higher possibility of developing acute respiratory distress syndrome (ARDS), as well as extra-pulmonary diseases [14]. As of November 30, 2020, the SARS-CoV-1, MERS-CoV, and SARS-CoV-2 pandemics have a mortality of 9.56%, 35.37%, and 2.34%, respectively [15]. 

The rapid expansion of the three outbreaks in the last two decades, attributable to CoVs, which are known to possess zoonotic origin, highlights the capability of CoVs to overcome species-specific barriers, generating epidemic and pandemic diseases with a great impact on human health [7]. The zoonotic reservoir of deadly viruses represents a threat of spillover zoonosis [2].

## 2. SARS-CoV-2 Genome Organization

**Figure 1:**
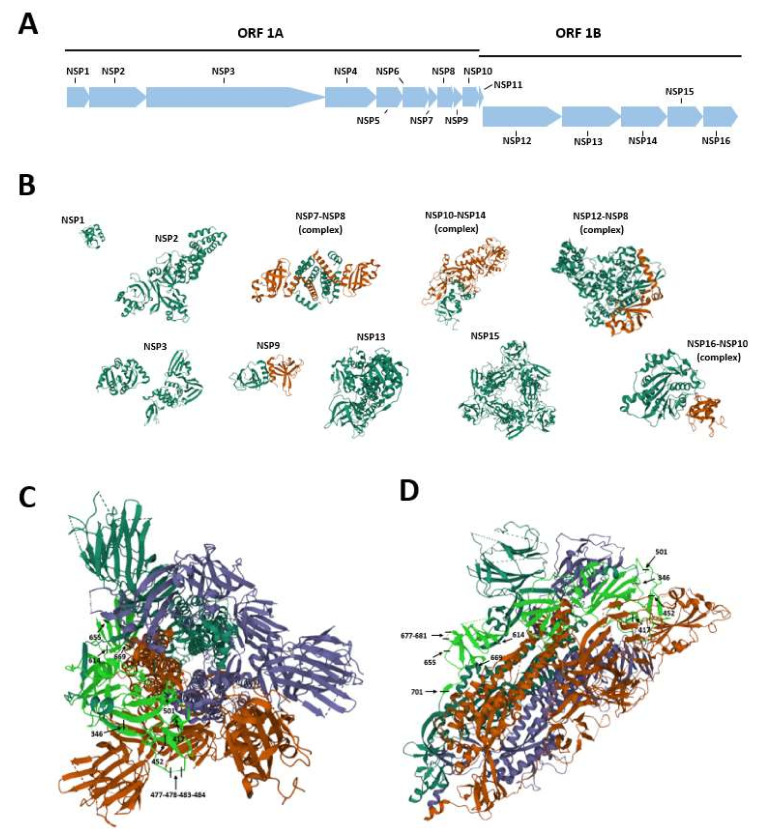
Known structures for SARS-CoV-2 proteins and mutation position of SARS-CoV-2 spike protein. (**A**) Schematic representation of genomic organization of SARS-CoV-2. (**B**) When available, the three-dimensional (3D) structure models were obtained from Protein Data Bank (PDB) database (https://www.rcsb.org/, accessed 11 August 2021). A cartoon representation for each available protein and/or protein complexes is shown. (**C**,**D**) Structural domain and mutation position of SARS-CoV-2 spike protein. The 3D structure of SARS-CoV-2 spike protein (6VYB, RCSB Protein Data Bank) is shown in up (**C**) and down (**D**) conformations. SARS-CoV-2 spike regions carrying mutations are RDB and SD1-2_S1-S2_S2 regions (labeled in green). (**C**) R346K, K417T, K417N, L452R, N501Y, D614G, H655Y, and G669S mutations are shown in the up conformation, while A701V mutation is not observable with this protein orientation. (**D**) R346K, K417T, K417N, L452R, N501Y, D614G, H655Y, G669S, and A701V mutations are observable in the down conformation. Due to protein orientation reasons, the localization of S477N, T478K, V483A, E484K, E484Q, Q677H, P681H, and P681R mutations is not visible with both up and down conformations.

The SARS-CoV-2 genome is a 26–32 kilobase (kb) non-segmented positive-sense single-stranded RNA [16,17]. The SARS-CoV-2 genome organization is similar to that of other CoVs [18,19]. The SARS-CoV-2 genome presents a total of 13–15 open reading frames (ORFs), including 12 functional ORFs, with 32–43% in G + C content [2,20]. The two untranslated regions (UTRs), 5′ UTR and 3′ UTR, play a role in both inter- and intra-molecular interactions by mediating RNA–RNA interactions, as well as the binding between viral and cellular host proteins [21]. In addition, the SARS-CoV-2 genome is capable of interacting with several host microRNAs (miRNAs) [22], which are small single-stranded non-coding RNA molecules [23]. Approximately two-thirds of the genome consists of two overlapping ORFs, named ORF-1a and -1b, encoding for the replicase polyproteins pp1a and pp1ab [24]. These proteins undergo autoproteolytic activity with the assistance of papain-like proteinase (PLpro) and 3C-like main protease (3CLpro or Mpro) to form 16 non-structural proteins (NSP1-16) (Table 1, Figure 1A,B) [25,26]. NSP1, -2, and -3, essential for viral replication, are produced by PLpro, which cleavages the replicase polyprotein at the N-terminus [27,28]. 

Lastly, the Mpro protein autoproteolytically divides pp1ab to produce mature viral enzymes and cleaved downstream nsps to release NSP4-NSP16 proteins (Figure 1A,B) [29]. 

The downstream region of ORF1b contains ORF sequences, which encode for four major structural proteins [30], i.e., spike (S) glycoprotein and envelope (E), membrane (M), and nucleoprotein (N) proteins [16,31]. The organization is 5′-leader-UTR-replicase/transcriptase- spike (S)-envelope (E)-membrane (M)-nucleocapsid (N)- 3′UTR-poly (A) tail [19]. These genes are located at ORFs 10 and 11 on the first one-third of the genome near the 30-end encode for these structural proteins [25]. These proteins are indispensable for viral maintenance and replication. Specifically, S, M, and E proteins are embedded to form the viral envelope [32]. The S glycoprotein is a type I membrane glycoprotein and constitutes virus peplomers. This protein is a key factor for SARS-CoV-2 infection and pathogenesis [33]. The M protein is the most abundant structural glycoprotein [34,35], playing a role in the transport of nutrients across the cell membrane and giving a shape to the virus particle [34]. The N protein binds, stabilizes, and protects the viral genome [36]. Furthermore, the E protein is essential in viral release and assembly during the viral cycle [34]. The remaining one-third of the SARS-CoV-2 genome encodes for accessory and structural proteins [35]. Virus particles can be spherical or pleomorphic with a diameter of about 60–140 nm. 

## 3. SARS-CoV-2 Spike Protein Structure and Functions

The SARS-CoV-2 spike S glycoprotein plays pivotal roles in viral attachment, fusion, and entry to the cell, and it is important in the determination of the virus tropism and host spectrum [37]. The S protein is a class I fusion protein (Figure 1C,D), 1273 amino acids (a.a.) long, and organized in trimers, which gives the crown-like shape to the virus. This conformation is typical of CoVs [38], where the S protein protrudes from the viral envelope. The trimeric class I fusion protein S consists of three subunits, known as the ectodomain, single-pass transmembrane anchor, and intracellular C-terminal tail. The ectodomain can be further divided into an (i) S1 domain where the receptor binding domain (RBD) is located and an (ii) S2 domain which mediates the viral fusion with the host cell membrane [33]. S trimers bind to the human cellular receptor angiotensin-converting enzyme 2 (hACE2) and, thus, allow the binding and entry of the virus into the host cell [39]. These trimers can be divided into two domains, named S1 and S2, with different functions. S1, located at the 14-684 a.a. position of the S protein, represents the globular part of the protein and possesses the region involved in the interaction with ACE2, the RBD domain, spanning from a.a. residues 331 to 524 [40]. RBD is determinant in the virus–receptor interaction [41]. S2, located within the 686-1273 a.a. positions of the S protein, forms the stem of the protein and contains the fusion machinery, or fusion peptide [42]. This domain comprises two heptad repeats (HR1 and HR2) that allow the formation of a six-helix bundle structure, which participates in the process of fusion and the release of the viral genome into the host cell. Furthermore, S2 contains a transmembrane domain that anchors the S protein into the cell membrane and a small cytosolic tail [42]. 

Several post-translational modifications have been found in the SARS-CoV-2 S protein, such as a unique N- and O-linked glycosylation pattern, which has not been found in SARS and MERS homologs. This pattern allows SARS-CoV-2 to evade the host immune system. Walls et al. demonstrated that the S protein contains 22 N-linked glycosylation (or sequons) [43], with 20 sequons conserved in SARS-CoV-1 [44]. Subsequently, O-linked glycosylations have also been identified, particularly at residues Thr323 and Ser325, which fall in the RBD within S1 [45]. These types of post-translational modifications on the S protein play a key role in (i) the proper protein folding; (ii) viral immune system evasion; and (iii) S protein priming/activation by host proteases, that is, S1/S2 cleavage [37,44]. Therefore, SARS-CoV-2 S protein recognition and binding to the ACE2 receptor occur through binding with the RBD [40], which possesses a 70% homology with that of SARS-CoV-1 [46]. Indeed, as for that of SARS-CoV-2, the SARS-CoV-1 S protein also interacts with the ACE2 receptor but with a significantly weaker affinity [40]. This difference, despite the well-known S protein sequence homology between the two CoVs, is due to a.a. substitutions established precisely in the RBD region, such as Val404 substitution for Lys417, but also to the Tyr442→Leu455, Leu443→Phe456, Phe460→Tyr473, and Asn479→Gln493 substitutions. In addition, the Arg426→Asn439, Tyr484→Gln498, and Thr487→Asn501 changes can be observed in the N-terminus (N-ter) region of the RBD, while in the C-terminus (C-ter) region, the substitution of Leu472 into Phe486 is present [47]. In addition to these substitutions, Ortega and colleagues highlighted the importance of two capping loops located at the RBD. These loops confer high electrostatic interaction potential, thereby providing the S protein with a high binding affinity to the ACE2 receptor. It has also been reported that these loops are longer in SARS-CoV-2 than in SARS-CoV-1. This might explain the higher affinity for ACE2 of the former compared to the latter [46]. 

Other studies, which focused on the binding between the C-ter portion of S (RBD) and the ACE2 receptor, confirmed that SARS-CoV-2 can establish additional weak (such as H and Van Der Waals bonds) and strong (such as aromatic–aromatic and ionic bonds) interactions than SARS-CoV-1 [33]. These chemical characteristics are due to a larger contact area between the viral protein and the receptor [33]. 

The binding to hACE2 receptor, which physiologically participates as a negative regulator in the renin–angiotensin system, appears to be equally important in the viral spread/entry [48]. hACE2 consists of a type I integral membrane glycoprotein with zinc metalloprotease function and a single catalytic domain [49]. In addition, the receptor as an enzyme degrades angiotensin II, a potent vasoconstrictor, into Ang (1–7), a vasodilating molecule. hACE2 is distributed in different body districts, including kidneys, heart, testis, and lungs. In the respiratory tract, it is expressed in human epithelial alveolar cells, where it favors the SARS-CoV-2 infection [48]. Furthermore, similarly to other genes [50,51,52,53], the hACE2 gene is under epigenetic regulation by DNA methylation [54]. Recent findings indicate that its improper methylation might increase SARS-CoV-2 susceptibility [54].

Cryoelectron microscopy analyses have shown that the binding between ACE2 and the S trimers provides conformational changes of the viral protein [55]. In particular, a change from a metastable pre-fusion to a stable post-fusion state occurs, allowing the virus to fuse with the cell membrane and enter into the host cell. These are so-called hinge-like conformational movements of RBD that transiently expose (up conformation) or hide (down conformation) it. It seems that the predominant conformational state presents one of the three RBDs rotated to the up position [56,57]. It has also been suggested that these up/down movements can be induced by a pH change in the surrounding environment. In particular, this biochemical process might occur during the entry of SARS-CoV-2 into the endosome following the cleavage via the protease transmembrane serine protease 2 (TMPRSS2), thereby leading to an all-down conformation that serves as a camouflage mechanism against the host immune system [58]. TMPRSS2 is a protease widely expressed in airway epithelial cells [59].

The following step of S glycoprotein–ACE2 binding is characterized by a series of proteolytic cuts of the spike protein mediated by host cell proteases that result in the initiation of the fusion process with the cell membrane. These cuts occur at different protein sites, such as S1/S2 and S2’ sites, and lead to the priming/activation of the S glycoprotein [60]. However, this complex process has not yet been fully understood. Initially, TMPRSS2 was indicated as a pre-activating enzyme, acting at the S1/S2 site. However, the identification of a furin polybasic cleavage site due to the insertion, just upstream of the S1/S2 site, of four PRRAR a.a. residues suggested the possibility of proteolytic processing by furins and furin-like proteases, which are ubiquitous in the human body [59,61]. In lung cells, this furin cleavage site, which is located at the S1/S2 region, is cleaved by furins at first (priming), and then protein activation occurs by TMPRSS2 and/or even cathepsin L [62,63]. In addition, it has also been reported that 2′ is not a furin cleavage site being cleaved by TMPRSS2 [63], while the RRAR sequence within the S1/S2 site is a multibasic furin cleavage site; therefore, it can also be cleaved by furins. 

### Summary

In summary, the SARS-CoV-2 S glycoprotein possesses a number of characteristics that improve virus efficiency in entering into the host cells [64]. These characteristics comprise a high affinity of RBD for the hACE2 receptor. Moreover, the SARS-CoV-2 S glycoprotein undergoes several conformational changes due to (i) interaction with the ACE2 receptor; (ii) pH of the surrounding environment; and/or (iii) activation by various host proteases, in particular pre-activation by furins. Overall, it is clear that the SARS-CoV-2 S protein possesses peculiar characteristics that have made the virus strongly affine to respiratory cell receptors and human proteases, allowing rapid human-to-human transmission.

## 4. SARS-CoV-2 Life Cycle

The binding between the S glycoprotein and the ACE2 receptor on the surface of the host cell [2,33,65] represents the initial step for SARS-CoV-2 infection [47,66,67]

In humans, the hACE2 receptor is expressed in almost all tissues, whilst being abundant in lungs and in the nasal and oral mucosa [68]. hACE2 is widely expressed on the epithelial cells of the trachea, bronchi, bronchial serous glands, and alveoli, as well as alveolar monocytes and macrophages [68]. After host protease processing, the region of the S2 domain named fusion peptide undergoes conformational changes and penetrates into the cellular membrane allowing SARS-CoV-2 fusion and entry into the host cell [69]. The host cell proteases cleave the S glycoprotein at the S1–S2 boundary, thereby allowing virus entry into the host cell. Indeed, S2 fuses the host and viral membranes and permits the entry of the viral genome into the host cell [33]. Additionally, while the entry of SARS-CoV-2 occurs through hACE2, the S glycoprotein priming is mediated by endosomal cysteine proteases cathepsin B and L (CatB/L) and TMPRSS2 [65]. Specifically, TMPRSS2 is a target of the alpha-1 antitrypsin (AAT) protein, which is one of the major serum proteins involved in anti-inflammatory processes [70,71]. AAT has also been considered for anti-COVID-19 therapy [72].

**Figure 2:**
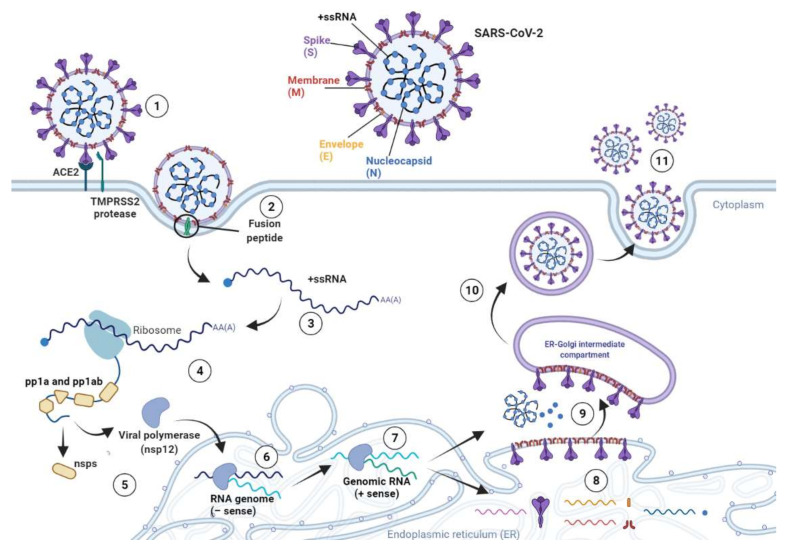
SARS-CoV-2 life cycle. (**1**) Binding between S glycoprotein and ACE2 receptor on the surface of host cell. (**2**) The fusion peptide undergoes conformational changes allowing SARS-CoV-2 fusion and entry into the cell cytoplasm. (**3**) Release of SARS-CoV-2 single-stranded positive RNA genome. (**4**) Viral genome is immediately transcribed by host cell ribosomes. (**5**) The translated RNA encodes polyproteins (pp1a and pp1ab) and the viral RNA-dependent RNA polymerase NSP12. (**6**) NSP12 produces full-length negative-sense copies of SARS-CoV-2 RNA. (**7**) The negative-sense RNA genome is employed as a template for generating the new positive-sense genomes. (**8**) The translation of the viral RNA occurs in the endoplasmic reticulum of host cells and leads to the synthesis of structural proteins. (**9**) Structural proteins move into the Golgi intermediate compartment where viral assembly occurs. (**10**) The mature viral progeny germinates from the intermediate compartment of the Golgi, and it is released as secretory vesicles. (**11**) SARS-CoV-2 virions are secreted by exocytosis.

Following the fusion between SARS-CoV-2 and the host cell membrane, the removal of the viral nucleocapsid protein allows the viral genome to be released into the cellular cytoplasm (Figure 2) [73]. 

Then, the viral genome acts as an mRNA being immediately transcribed by host cell ribosomes that translates two-thirds of this viral ribonucleic sequence. The translated RNA, corresponding to ORF1a and ORF1b, encodes pp1a and pp1ab [74,75]. These polyproteins are subsequentially processed to obtain functional non-structural proteins 1–16 (Figure 1), which are essential for the replicase–transcriptase complex formation. 

The synthesis of SARS-CoV-2 proteins is associated with the replication of the viral genome (Figure 2) [76]. The RNA-dependent RNA polymerase NSP12 produces full-length negative-sense copies of SARS-CoV-2 RNA, which are afterward employed as templates for generating new positive-sense genomes. Then, newly synthesized RNA strands act as a genome to generate the viral progeny [24,26,76]. Furthermore, other ORF1a- and ORF1b-derived mRNAs are also translated into S, E, M, and N proteins along with accessory proteins ORF3a an ORF9b [76]. The translation of viral RNA occurs in the endoplasmic reticulum of host cells and leads to the synthesis of S, E, and M structural proteins, which move through the secretory pathway into the Golgi intermediate compartment [74,76]. Here, the new RNA genome is packed by the viral N protein and then shaped to form a helical nucleocapsid [24,26]. Subsequently, the M protein attends the assembly of the virion and the incorporation of the nucleocapsid, envelope, and S glycoproteins into virus particles [26]. When virion assembly is completed, the viral progeny germinates from the intermediate compartment of the Golgi, and it is released as secretory vesicles. Lastly, SARS-CoV-2 virions are secreted from the cell by exocytosis (Figure 2) [24,76].

## 5. SARS-CoV-2 Transmission from Animals to Humans

### 5.1. The Spillover Theory

SARS-CoV-2 originated in animals and transmitted to humans, afterward exhibiting human-to-human transmission [77,78]. Two possible scenarios have been inferred to date. In the first, SARS-CoV-2 might be transmitted to humans where it suddenly underwent a natural selection. In the second, a natural selection of the virus in an animal host may have occurred before its transmission to humans [79]. In agreement with the spillover theory, a number of studies attempted to identify a putative intermediate host that facilitated SARS-CoV-2 infection of the human population [78,80,81,82]. The most recent theories assumed that SARS-CoV-2 has been transmitted from bats to humans through an intermediate animal host. However, direct contact having occurred in a so-called wet market in Wuhan, a marketplace selling fresh and wildlife meat, cannot be excluded. In these such marketplaces, a large variety of wildlife species are slaughtered and sold, whilst operators might be in constant risk of contamination via blood/fresh meat contact. This animal-to-human transmission modality has been theorized for polyomavirus simian virus 40 (SV40), where it has been described that inhabitants of villages and/or workers attending to monkeys in zoos/animal facilities were more prone to SV40 infection [83]. In this context, the influence of the wet markets on the early transmission dynamics of SARS-CoV-2 from bats, if present, should be investigated. Although bats have been identified as SARS-CoV-2 natural hosts, the intermediate host remains to be identified [84]. The identification of the animal reservoir, if present, is crucial to interrupt the transmission chain of SARS-CoV-2. 

### 5.2. Role of Bats

A number of different in silico studies have been conducted to identify the intermediate animal host. Comparisons/phylogenetic analyses of both S protein strains and the full-length genomes from SARS-CoV-2 and a variety of CoVs have been performed. SARS-CoV-2 shows extensive sequence homology with SARS-CoV-1. In addition, SARS-CoV-2 and bat CoVs share 89.2% homology, thereby suggesting the possibility that SARS-CoV-2 may have originated from bats [80,81,82,85]. A similar conclusion has previously been drawn for both SARS-CoV-1 and MERS-CoV [30]. SARS-CoV-2 also presents 97.4% similarity to CoVs from pangolins, thus highlighting the latter as possible intermediate hosts of the virus [86]. The hypothesis that SARS-CoV-2 may also have been transmitted from pangolins to humans following mutational events on pangolin CoV strains cannot be ruled out. Interestingly, according to a full-length genome sequence analyses, it was suggested that SARS-CoV-2 may have arisen from a CoV of unknown origin or the bat CoV as a recombinant mutant virus [78]. Then, at some point of bat infection, SARS-CoV-2 underwent mutational events on its strain prior to human transmission, acquiring its ability to infect humans [81]. The presence of mutations in both the S and N proteins that distinguish SARS-CoV-2 from bat SARS-like CoVs corroborates this theory.

Notably, the phylogenetic analysis of the whole full-length strains of SARS-CoV-2 and other bat CoV strains indicate that the full-length strains were roughly similar, while the region encoding for the S glycoprotein was particularly divergent. Therefore, a number of works focused on the S glycoprotein with particular attention for the RBD region located at the S1 domain [87]. Indeed, SARS-CoV-2 exerts its infectious potential through the binding between the RBD and the ACE2 receptor [46,88]. Thus, RBD is the biologically active region of the S glycoprotein, and it is therefore under positive selection or host driven [87]. A large amount of computational data on S glycoprotein/RBD/S1 has been therefore generated. It has been reported that SARS-CoV-2 S1 exhibits a low degree of homology with the two so-called bat-derived ancestors, i.e., bat-SLCoVZC45 and bat-SL-CoVZXC21 [89]. Contrariwise, S2 exhibits a high degree of similarity [89]. Moreover, although SARS-CoV-2 and SARS-CoV-1 belong to two different clades, the S glycoprotein of these two CoVs exhibits a certain degree of linear/conformational similarity. These two CoVs share about 50 amino acid residues in the S1 domain. Moreover, the three-dimensional (3D) homology models of SARS-CoV-2 RBD with that of SARS-CoV-1 indicate a high degree of similarity in the conformation/structure of the receptor binding motif. Consistently, a growing body of hypotheses suggested that animal–human transmission of SARS-CoV-2 has been due to recombination events throughout the viral genomes, particularly in the RBD coding region of the S glycoprotein. These recombination events between the bat-derived SL-CoVZC45 and SL-CoVZXC21 leading to zoonotic SARS-CoV-2 might have taken place in a still unknown intermediate host [89]. 

Although the RBD is the most SARS-CoV-2 S glycoprotein-variable region [79], it is important to point out that this domain must be considered in the context of the entire S glycoprotein and not as a separate entity. Its coding sequence is not independent of the rest of the S coding gene. Moreover, 3D conformation of the RBD depends mainly on that of the whole S glycoprotein, which in turn depends on the specific interactions among all a.a. residues within the entire protein. Therefore, in silico models of the RBD, which is essentially a short sequence in the context of the whole S glycoprotein, give poor information and cannot provide a clear indication of complete similarity [87]. 

### 5.3. Possibility of Pangolins as the Intermediate Host 

In silico analyses conducted on sequence similarities between the RBD of the S glycoproteins of the Malayan or Javan pangolin (Manis javanica) and *R. affinis* Sarbecoviruses (bat CoVs) led to the proposal of pangolins as the intermediate animal host [90,91,92,93,94,95,96]. A number of computational models predicted recombination events between pangolin CoVs and Sarbecoviruses [87]. This evidence has also been corroborated by the fact that pangolins have already been identified as the host of Sarbecoviruses related to SARS-CoV-2, and they are highly considered in the traditional Chinese medicine market [80,88,96,97,98,99]. However, it is worth recalling that these conclusions have been postulated on purely computational data [87]. Although these in silico analyses predicted recombination events between CoVs from pangolins and Sarbecoviruses, these two species, bats and pangolins, not only live in different geographical regions but also show a different ecology/biology, making such a recombination event physically unlikely. However, it is important to point out that these two species can be simultaneously found in wet markets. An additional recent in-depth genomic analysis indicated that CoVs related to SARS-CoV-2 have been circulating in bats for decades [97], with the lack of recombination events being reported. Other studies confirmed this theory [87]. In addition, the low binding affinity between the SARS-CoV-2 RBD and the pangolin ACE2 receptor later confirmed this conclusion [87]. Previous data indicate that SARS-CoV-2-like Sarbecoviruses infect Rhinolophus bats worldwide [87]. The comparative analysis of the complete RNA-dependent RNA polymerase (RdRp) sequence revealed that the Sarbecovirus from pangolins is closer to a Kenyan Rhinolophus bat, whilst SARS-CoV-2 appears to belong to a different branch compared to the Sarbecoviruses identified in pangolins [87]. Despite the reduced sequence content allowing comparisons, similar information can also be provided by the S coding gene. Sequence analyses of the S gene indicate that (i) SARS-CoV-2 and Sarbecoviruses from pangolins fall into different clusters, which are separated by high bootstraps; and (ii) SARS-CoV-2 is closer to *R. affinis* Sarbecovirus. In summary, the role of the pangolin as the intermediate host in the transmission of SARS CoV-2 from bats to humans seems to be unlikely [87]. This conclusion is supported by the results of another study [98], which ultimately ruled out the pangolin as an animal intermediate host.

### 5.4. Role of S Protein Furin Cleavage Site

Several hypotheses are emerging in order to understand whether the S protein characteristics might somehow give information about which intermediate host, if any, enabled the spillover of SARS-CoV-2 into the human population. Attention has also been given to the furin cleavage site, which, shared by several beta-CoVs, has also been identified in various strains of feline coronavirus (FCoV) [99]. Based on structural and biochemical analyses, Wrobel and colleagues suggested that the furin cleavage site confers an advantage for SARS-CoV-2 to infect humans, compared to that of bat-CoV RaTG13, by facilitating the conformation change of the S protein to the open form and thus favoring binding to the hACE2 receptor [100]. Indeed, alignments with other CoVs indicated that this solvent-exposed PRRAR↓SV site may represent a gain of function of SARS-CoV-2 compared with other beta-CoVs, such as SARS-CoV-1. Contrariwise, β-CoVs of the b lineage do not present such sequences [101]. It is still unclear whether the furin site is derived from a common ancestor of SARS-CoV-2 or was acquired later by spontaneous mutations. These mutations could be determined by serial passages in intermediate animals or even in in vitro/in vivo laboratory experiments [102]. Indeed, it has been reported that SARS-CoV-2 in vitro propagation may occur as a result of viral insertions and/or deletions [103]. However, due to the lack of data, this scenario is unlikely. 

### 5.5. ACE2 Receptor and Intermediate Animal Host Identification

Recognition/binding between the S glycoprotein and the ACE2 receptor is a key determinant for SARS-CoV-2–host interaction range, tissue tropism, and viral pathogenesis. This binding represents the initial step for virus entry into cells [47,67,68]. In humans, the SARS-CoV-2 S glycoprotein interacts with the hACE2 receptor with high affinity, thereby giving SARS-CoV-2 the ability to infect humans along with high human-to-human transmission potential [88]. Indeed, hACE2 has been described to be expressed in human cells [89,104], and a study performed on HeLa cells expressing hACE2 reported that the SARS-CoV-2 S glycoprotein binds to hACE2 with high affinity [89,104]. It has also been reported that the binding affinity of SARS-CoV-2 S glycoprotein to the hACE2 receptor is higher than that of SARS-CoV-1 [46,88]. This high binding potential most likely implies an interspecies transmissibility of SARS-CoV-2 [105] as well as very rapid human-to-human transmission of this retrovirus [46,88]. 

Previous data indicate that the ACE2 receptor is expressed in a large number of mammalian species and birds, and it has been reported that the SARS-CoV-2 S glycoprotein recognizes the ACE2 receptor from a variety of animal species. Taking these aspects into account, a series of investigations have been conducted to define the zoonotic potential of this ligand–receptor interaction, with particular attention paid to the ACE2 receptor sequence/conformation. Computational analyses of the a.a. sequence and 3D models of hACE2 revealed that this receptor possesses a.a. residues located at positions 30–41, 82–93, and 353–358, which play a fundamental role in the receptor–S protein interaction [56,106]. Therefore, with the aim of identifying the putative intermediate animal host, a large number of computational analyses/studies have been performed to identify a.a. residues within the ACE2 sequence, across different species, being required to specifically bind the SARS-CoV-2 S protein [67,94,107,108,109,110,111,112]. Several a.a. located within ACE2 have been reported as determinant in this binding. In particular, a.a. K31, N82, N90, and K353 within the ACE2 receptor sequence from Rhinolophus sinicus (Chinese horseshoe bat) are considered to be favorable a.a. interacting with the SARS-CoV-2 S protein. Likewise, the ACE2 receptor from Manis javanica (Sunda pangolin) is characterized by K31, Y41, N82, N90, and K353, which are favorable a.a. for SARS-CoV-2. In humans, hACE2 carries several key a.a. that interact with the SARS-CoV-2 S glycoprotein, namely K31, Y41, N90, and K353 [113,114]. To determine the SARS-CoV-2 intermediate host, a phylogenetic analysis has been performed on a total of 207 sequences of ACE2 from 198 mammalian species [113,114]. The study reported 24 key a.a. residues within the hACE2 sequence for binding the S protein of SARS-CoV-2. In addition, the study also reported that ACE2 from bats underwent positive selection, and the species possessing similar a.a. residues to the key human a.a. residues might potentially be susceptible SARS-CoV-2 hosts [113,114]. Homology modeling of the SARS-CoV-2 S glycoprotein and ACE2 from different species predicted that this viral protein could potentially interact with the receptor belonging to an incredibly high number of species, including primates, such as Old World monkeys, orangutans, and baboons; mustelids and civets; cats; tortoise; pangolins; pigs; ferrets; horses; sheep; suines; cows; rabbits; red foxes; dogs; several species of bats; and hamsters [67,91,107,108,109,110,111,114]. However, the extent of this interaction varies according to the considered species. For instance, three species of pangolin, namely Manis pentadactyla, M. Javanica, and Phataginus tricuspis carry ACE2 receptors with a low binding affinity for the SARS-CoV-2 S glycoprotein [79].

Evaluating the expression levels of ACE2 in different mammalians and comparing their ability to bind to the SARS-CoV-2 S glycoprotein could represent an alternative approach for the identification of the intermediate animal host [114,115]. In the effort to identify the intermediate animal host of SARS-CoV-2, the receptor activity of ACE2 from 14 mammal species has recently been exanimated through a binding assay using pseudo-typed lentiviruses expressing the wild-type or furin cleavage-deficient S glycoprotein of SARS-CoV-2 [110]. It turned out that human and rhesus monkey ACE2s strongly bound to the SARS-CoV-2 S glycoprotein with the highest affinity, whilst rat/mouse ACE2 had the lowest [110]. Interestingly, among non-primates, rabbit and pangolin ACE2s have been found to bind strongly to the S1 subunit of the SARS-CoV-2 S glycoprotein [110].

### 5.6. Role of Other Intermediate Hosts 

CoVs, similarly to other infectious agents [116,117,118,119], can infect both animals and humans [3,6,7]. Different animal species are reported as hosting CoVs, such as horses, cattle, camels, swine, rabbits, rodents, cats, dogs, bats, birds, snakes, minks, and other wild animals. These potential intermediate hosts allow the maintenance of pathogen circulation, promoting the possibility of human infection. Moreover, in intermediate hosts, CoVs could differentiate and generate new viral progeny from the parental strains and then change pathogenicity and host range [7]. While SARS-CoV-1 and MERS-CoV are recognized as responsible for human infection and present palm civet cat and dromedary camels, respectively, as intermediate hosts, the SARS-CoV-2 intermediate host is still unknown [120,121,122]. Since elective vaccines and antiviral therapies in veterinary medicine against animal infection could be prophylactic and therapeutical strategies against SARS-CoV-2, researchers are investigating which animals may act as SARS-CoV-2 intermediate hosts [7]. Different animals were first considered, particularly those that have possible close contact with infected humans. Actually, some studies have experimentally demonstrated that SARS-CoV-2 recognizes ACE2 from rabbits, dogs, pigs, ferrets, hamsters, rhesus monkeys, tree shrews, marmosets, civets, cats, and pangolins [108,123,124,125]. The efficiency of ACE2 usage in different animals could indicate their susceptibility to SARS-CoV-2 infection [123]. A serological study detected SARS-CoV-2 neutralizing antibodies in cats in Wuhan after the COVID-19 outbreak, providing SARS-CoV-2 infection in cats [123,126]. Moreover, a cat from Belgium and another one from Hong Kong tested SARS-CoV-2 positive [120]. In cats that live with SARS-CoV-2-positive owners, a higher titer of neutralizing antibodies was seen [120,127]. However, cat-to-human transmission is uncertain, while cats are able to transmit the virus to other cats without showing any signs of illness [110,111,122,128,129]. In zoo settings, animals that had contact with infected zookeepers were tested: in the Bronx Zoo of New York City, a Malayan tiger tested positive for SARS-CoV-2, not showing signs of mild respiratory illness. SARS-CoV-2 was also detected in a zoo lion, maybe infected by its zookeeper [111,122,130]. In Hong Kong, two dogs from households with human cases of COVID-19 had natural SARS-CoV-2 infection evidenced by virological and serological testing. One case was reported in a German Shepherd dog and the other one was a 17-year-old Pomeranian dog in which RT-PCR in both oral and nasal samples resulted positive, while a serological test gave positive results in later stages. Both dogs had a weak infection and were asymptomatic. Currently, findings are suggestive of an infection caused by human-to-dog transmission, and there is no evidence of dog-to-human transmission [110,122,131,132]. According to a recent experimental infection, pigs are not susceptible to SARS-CoV-2 [7,108]. However, they can be infected by influenza and bat CoVs, and the possibility of evolution of a new virus from influenza and CoVs cannot be excluded [122,133,134].

In both Denmark and the Netherlands, an outbreak of SARS-CoV-2 infection in minks was documented: while most infected minks had mild symptoms, some of them died of interstitial pneumonia and severe respiratory distress [110,127,135]. 

Phylogenetic analysis shows that spillover can occur from mink to humans in mink farms, but further research will be needed to determine whether these animals could be reservoirs of SARS-CoV-2 [125]. In contrast to minks, other infected animals described appear to not be able to transmit the infection to humans, and they are considered to be final hosts.

SARS-CoV-2 has, with high probability, a zoonotic origin, since highly related sequences were detected in bats, but the role of animals in the epidemiology is still largely unknown. Specific intermediate hosts have not been clearly proven yet. On the basis of phylogenetic data, it is unlikely that SARS-CoV-2 passed directly from bats to humans without an intermediate host [81]. It was assumed that SARS-CoV-2 was initially transmitted from animals to humans, and transmission continued via human-to-human transmission [78,120]. Indeed, human-to-human transmission occurs more easily than animal-to-human transmission [128]. SARS-CoV-2 has been detected in different animals, confirming SARS-CoV-2 infection could be considered an anthropozoonosis, defined as human-to-animal transmission. However, one million human SARS-CoV-2 infections are reported to be exclusively through human-to-human transmission, thus not meeting the World Health Organization (WHO) definition of zoonosis. The WHO defines a zoonosis as “any infection that is naturally transmissible from vertebrate animals to humans”: the infection is maintained in an animal population, which is the source of human infection [129]. Considering the Emergency Infectious Disease definition, it is defined as “Diseases that have newly appeared in a population or have existed, but are rapidly increasing in incidence or geographic range” [129,130].

**Figure 3:**
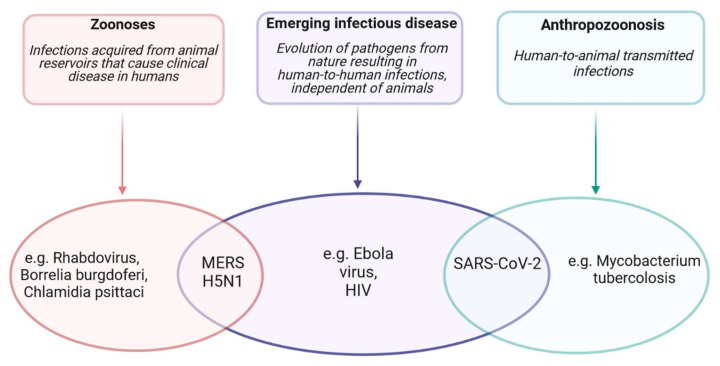
SARS-CoV-2 spillover to humans hypotheses. Spillover to humans can result from three different processes: zoonoses in which pathogens are transmitted from the animal reservoir directly (such as Rhabdovirus or Chlamydia psittaci) or indirectly (e.g., via vectors such as Borrelia burgdorferi) to humans causing disease. Emerging infectious diseases in which pathogens can evolve from animals and cause an emerging disease of human infection that is characterized by human-to-human transmission independent of animals (e.g., Ebola virus and HIV). Anthropozoonosis which is an infection transmitted from human to animal (such as Mycobacterium tuberculosis infection). Some pathogens may fall into more than one category: MERS and H5N1 are both zoonoses and emerging infectious diseases. The SARS-CoV-2 infection has characteristics of both an emerging infectious disease and an anthropozoonosis, but from this point of view, it is not considered a zoonosis because no animal is found as a reservoir, and human infection can only occur by human-to-human transmission.

COVID-19 could be considered an emerging infectious disease of probable animal origin caused by the spillover mechanism. Spillover is the natural animal–new host transmission of a pathogen. Infection in the new host may result in a dead end (in the final hosts) or may lead to anthropozoonotic transmission or spread through secondary epidemiological cycles [129]. Considering the animal origin, there are two hypotheses for the emergence of SARS-CoV-2: a natural selection of the virus in an animal host before jumping to humans or a natural selection of the virus in humans after zoonotic transmission [120,129]. Figure 3 explains an example of zoonoses, anthropozoonoses, and emerging infectious diseases, defining SARS-CoV-2 as an emerging infectious disease of animal origin characterized by the features of anthropozoonoses (human-to-animal infection).

### 5.7. Summary

At present, there is no evidence that SARS-CoV-2 is a directly or indirectly transmitted zoonotic disease, but it is certainly transmitted from human to human without requiring maintenance in a separate reservoir species as in other infectious diseases [129]. From this point of view, SARS-CoV-2 infection is no longer considered a pure zoonosis but an emerging human infectious agent with a probable animal origin that can be transmitted from humans to certain animal species through close contact.

## 6. Diagnostics for SARS-CoV-2 Infection 

One of the main steps in managing COVID-19 is the early, rapid, and accurate detection of SARS-CoV-2 infection. With the development of molecular biology techniques/protocols [131,132,133,134,135], the detection of SARS-CoV-2 is becoming more accurate and precise [136]. The gold standard for detecting SARS-CoV-2 is the real-time reverse transcription–polymerase chain reaction (RT-PCR) [136]. This molecular biology technique can potentially detect and quantify SARS-CoV-2 nucleic acids present in nasopharyngeal fluids as well as potentially in any human fluids/tissues. Accurate viral detection and viral RNA load quantification is paramount to contain the COVID-19 pandemic worldwide. Therefore, improving test sensitivity and specificity remains an urgent need [137]. A more sensitive technique is represented by the droplet digital PCR (ddPCR), which provides an accurate and reliable detection and quantification of viral nucleic acids as demonstrated for other viruses [138,139]. However, although several reports have demonstrated its high reliability in detecting SARS-CoV-2 nucleic acids [140], its use is far from the clinical routine. 

An additional diagnostic procedure for detecting SARS-CoV-2 infection is serological testing based on immunological assays, which complements the detection of viral RNA. The enzyme-linked immunosorbent assay (ELISA) can indicate past infection, which could also be exploited for therapeutic purposes as a prognostic tool [141]. The ELISA test can potentially detect IgG and/or IgM antibodies against the viral S protein in addition to other viral proteins [142]. Determining the response against SARS-CoV-2 proteins, this such assay may provide protection against subsequent viral exposure and can be used for patient monitoring [143]. 

On this basis, these techniques/protocols aimed at detecting SARS-CoV-2 infection are important in managing the COVID-19 pandemic. The early, rapid, and accurate detection for epidemiological evaluations as well as for patient monitoring is also a true and broad global therapeutic need [144]. Future experimental designs should provide the development of diagnostic/prognostic tools aimed at improving SARS-CoV-2 detection sensitivity and specificity [143]. 

## 7. Food and Drug Administration-Approved Antibody Therapy

Currently, a variety of therapeutic options are available for the treatment of COVID-19 disease and for the management of SARS-CoV-2 infection. Patients today have growing treatment options in the battle against this disease. In the last few months, the U.S. Food and Drug Administration (FDA) has issued a number of Emergency Use Authorizations (EUAs) for several monoclonal antibody treatments of mild and/or moderate COVID-19 in SARS-CoV-2-positive adults and pediatric patients, i.e., aged at least 12 years old and weighing at least 40 kg, and who are at high risk for progressing to severe COVID-19 and/or hospitalization. The current FDA-approved antibody therapeutic options are represented by the use of Actemra^®^ (tocilizumab), bamlanivimab and etesevimab, sotrovimab, bamlanivimab, and REGEN-COV (casirivimab and imdevimab), all available since approximately the end of 2020 (https://www.fda.gov/emergency-preparedness-and-response/mcm-legal-regulatory-and-policy-framework/emergency-use-authorization#coviddrugs, accessed 12 August 2021). All these therapeutic options obtained positive results in managing COVID-19 disease, while more therapies are currently being tested in clinical trials to evaluate whether they are safe and effective in the treatment of COVID-19.

## 8. From SARS-CoV-2 Entry to the Development of Cytokine Storm Syndrome 

**Figure 4:**
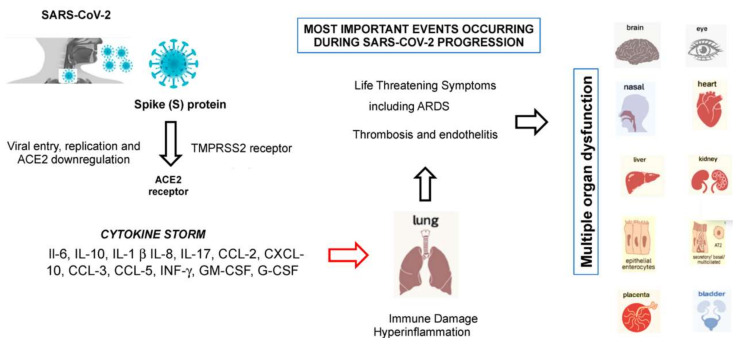
SARS-CoV-2 infection. Angiotensin-converting enzyme 2 (ACE2) expression is also found in respiratory and pulmonary tract cells (alveolar monocytes and macrophages), with the possibility of severe acute respiratory distress syndrome (ARDS) and in heart, kidneys, brain, endothelium, and liver, in which organ failure and thromboembolism may occur.

After membrane fusion, the viral RNA is released into the cytoplasm, facilitating its replication [12]. The abundant expression of the hACE2 receptor in type II alveolar cells promotes a rapid viral multiplication with subsequent destruction of the local alveolar wall. This process results in rapidly progressive and widespread alveolar damage with the occurrence of concomitant cytokine/chemokine cascades (Figure 4) [12]. In this setting, females seem to have fewer hACE2 receptors on the lung surface than males, and this could contribute, together with some genetic and hormonal factors, to give more protection from COVID-19 infection and make them less susceptible [145]. Studies are underway to support these hypotheses. 

Recent studies have demonstrated the virus entry mechanism, also clarifying the contribution of such a mechanism to the evasiveness of the virus against immune response, cell infectivity, and spreadability of the virus [64]. Basically, differently from other CoVs, SARS-CoV-2 entry is dependent on preactivation by proprotein convertase furin, thus reducing its need for target cell proteases. Such a mechanism allows SARS-CoV-2 to maintain efficient cell entry while evading immune surveillance, thus potentially contributing to the wide spread of the virus. 

The hACE2 receptor is also highly expressed on the luminal surface of intestinal epithelial cells, which may explain some initial disease manifestations, such as vomiting and diarrhea [146]. Thus, the intestine might be a major entry site for SARS-CoV-2. However, less than 10% of children with infection develop diarrhea and vomiting [147]. The infection of human gut epithelium has important implications for fecal–oral transmission and the containment of viral spread. Whether COVID-19 may have started with the consumption of bat food from the Wuhan market, the presumed location of the outbreak, is yet to be determined [148]. Moreover, hACE2 receptor tissue distribution in organs, such as the heart, kidney, endothelium, and retina, could explain the multi-organ dysfunction observed in patients. 

Accumulating evidence suggests that SARS-CoV-2 causes an inflammatory response in the lower airway, leading to lung injury [12]. Collectively, the virions first invade the respiratory mucosa and then trigger a powerful immune response in the lungs with the production of a cytokine storm, named cytokine storm syndrome (CSS), which hyperactivates a type-1 cellular T-helper response similar to that described in SARS and MERS [149]. CSS is an uncontrolled and often fatal systemic inflammatory response, resulting from the release of large amounts of pro-inflammatory cytokines, including IFN-α, IFN-γ, IL-1β, IL-6, IL-12, IL-17, IL-18, IL-33, TNF-α, and TGF β, and chemokines, including CCL2, CCL3, CCL5, CXCL8, CXCL9, and CXCL10. Cytokines/chemokines all contribute to the occurrence of ARDS and may lead to the most critical condition of COVID-19 patients, including death [149,150]. Recent evidence suggests that receptors of purinergic signaling, such as adenosine receptors [151], could be employed for pharmacological targeting to protect against myocardial injury caused by a cytokine storm in COVID-19 [152]. Other than lung epithelial cells, SARS-CoV-2 also infects macrophages and dendritic cells, which overstimulate and promote hyperinflammation. Most cytokines produced during SARS-CoV are secreted by macrophages and other mononuclear phagocytes. Therefore, macrophages could be a key target to stop the extensive lung damage caused by COVID-19 [150]. IL-17 is able to strengthen the inflammation response and to activate neutrophil cells that can migrate to the lung and are heavily involved in the pathogenesis of SARS-CoV-2 infection. Recently, some Italian researchers have shown that blocking IL-17 could provide a novel therapeutic strategy for COVID-19. 

CSS is an awful, life-threatening situation that can lead to harmful effects, including organ failure, tissue toxicity, loss of capillaries, and shock. The activation of platelets triggered by this cytokine activation can contribute to hypercoagulability, the cause of pulmonary thromboembolism [153], leading to death from respiratory failure. The most common cause of death among infected patients is severe respiratory failure. Patients with severe presentation, which in part resembles SARS-CoV-1 and/or MERS-CoV infections [154], may develop ARDS and require ICU admission, although oxygen therapy and assisted intubation may not be useful because of pulmonary and generalized venous thromboembolism [153].

Since the hACE2 receptor is also widely distributed in different organs, such as the heart, kidney, endothelium, and retina, this could explain the multi-organ dysfunction observed in COVID-19-affected patients. Therapeutic studies based on the rational use of anti-inflammatory drugs that directly inhibit the synthesis processes of inflammatory cytokines, e.g., inflammasome nucleotide-binding oligomerization domain (NOD)-like receptor family, pyrin domain-containing 3 (NLRP3) [155], are currently underway in these patients. Early control of CSS by immunomodulators and cytokine antagonists at an early stage, as well as the reduction of lung inflammatory cell infiltration, is the key to improving treatment success rates and reducing mortality rates in patients with COVID-19. Therefore, rational therapy should always include a careful evaluation of the cytokine’s profile of selected cohorts of symptomatic COVID-19 patients, especially those with severe pneumonia and in intensive care units (ICUs). 

## 9. The Impact of SARS-CoV-2 Variants and Their Role in Viral Infection

With global spreading of the SARS-CoV-2 infection, viral variants rapidly emerged in the population, prevailing on original strains found at the beginning of the pandemic [156,157]. Several mechanisms may be involved in the emergence of new mutations/variants, such as natural selection, persistent infections involving immunocompromised patients, chance events, host shifts, and mutation involving the proofreading function [158]. Some of the emerging variant mutations have been identified in immunocompromised patients with persistent viral shedding, in natural non-responders, or in patients previously treated with convalescent plasma or monoclonal antibodies against the S protein [159,160], suggesting an in vivo selection of mutations capable of escaping antibody response. 

One of the first identified variants is associated with the D614G mutation of the spike protein gene. This variant modifies the spike protein conformation, promoting the formation of an ACE2-binding fusion-competent state, allowing an increase in viral infectivity; its sensibility to neutralizing antibodies is not impaired [156,161,162,163]. 

According to the classification made by ECDC (European Centre for Disease Prevention and Control), updated on 24 May 2021 [164], SARS-CoV-2 variants can be divided into three categories: Variants of Concern (including B.1.1.7, B.1.1.7 + E484K, B.1.351, P.1, and B.1.617.2) (Table 2, Figure 1C,D), Variants of Interest (B.1.525, B.1.427/B.1.429, P.3, B.1.616, B.1.617.1, B.1.617.3, B.1.620, and B.1.621) (Table 3, Figure 1C,D), and variants under monitoring (B.1.214.2, A.23.1 + E484K, A.27, A.28, C.16, C.37, B.1.351 + P384L, B.1.351 + E516Q, B.1.1.7 + L452R, and B.1.1.7 + S494P).

Variants Of Concern (VOCs) are characterized by several mutations and deletions on the interesting spike protein, especially its RBD and its N-terminal domain (Table 2) [158,166]. 

One common mutation between identified VOCs B.1.1.7, B.1.351, and P.1 is N501Y, which probably allows an increased affinity between the SARS-CoV-2 RBD domain and the ACE2 receptor [167,168]. Planas et al. [166] demonstrated an improved binding capability of S proteins carrying the N501Y mutation to the soluble ACE2 protein when compared to the D614G-mutated strain of SARS-CoV-2.

Among the new variants, VOC 202012/01 (English variant) was identified for the first time in the UK between late summer and early autumn 2020. Gene sequencing of community diagnostic tests revealed a very rapid spreading of this variant, suggesting a selective advantage [169]. This variant belongs to the B.1.1.7 lineage, and it is characterized by several changes in the S protein when compared to the Wuhan original strain (archaic strain): N501Y, A570D, D614G, P681H, T716I, S982A, and D1119H mutations, but also deletions in residues 69–70 and 144. The most concerning variation is N501Y, responsible for increased affinity between the RBD and the ACE2 receptor and possibly capable of interrupting binding with some neutralizing antibodies [167]. VOC 202012/01 has an increased transmissibility; it is estimated to be 43–90% more infectious than other strains circulating during the first wave of the pandemic [170].

Calistri et al. [171] demonstrated that VOC 202012/01 infection is characterized by increased viral load and prolonged viral persistence compared to original strains. According to a work conducted by Davies et al. [172], mortality rates related to this variant infection represent an increased absolute risk in elderly people in comparison with the ancestral Wuhan strain; overall, the mortality rate following a positive community diagnostic test remains less than 1% in most individuals under 70 years old. 

VOC 20H/501Y.V2 originated in South Africa in October 2020 [168]. Within a month, this variant became the dominant strain in this region, suggesting high rates of transmissibility, although there was no evidence of increased virulence or disease severity [158]. The South African variant belongs to the B.1.351 lineage, and it is characterized by three mutations in the RBD domain of the spike protein: K417N, E484K, and N501Y. E484K and K417N are mutations of concern because they can partially compromise neutralizing antibodies generated by vaccination or previous SARS-CoV-2 infections, and they can decrease susceptibility to several monoclonal antibodies [172,173,174,175]. Pfizer and Moderna vaccines maintain their efficacy against lineage B.1.351, even if slightly reduced compared to that of the archaic strain [168,176]. Moreover, the Janssen monodose vaccine partially preserves its activity against this lineage [168], differently from the AstraZeneca (now Vaxzevria) vaccine, of which activity is particularly impaired [173].

P.1 (Brazilian variant) was discovered in January 2021 in Japan by analyzing nasopharyngeal swabs of travelers from the Amazon Forest, Brazil. This variant was later identified in Brazil, where it was the dominant strain [159]. The P.1 variant belongs to the B.1.1.28.1 lineage and contains 12 different mutations in the S protein, in particular the K417T, E484K, and N501Y mutations, which are located in the RBD domain. E484K, a common mutation with VOC 20H/501Y.V2, is of particular concern because it may impair the neutralizing capacity generated by convalescent plasma or plasma from vaccinated persons. Considering the similarity with VOC 20H/501Y.V2 and the presence of several other mutations, this variant is probably just as resistant, or probably even more so, to convalescent plasma or vaccination [158].

A study conducted by Funk et al. [177] compared clinical characteristics of the SARS-CoV-2 variants B.1.1.7, B.1.351, and P.1 to non-variant cases registered by seven European countries (Cyprus, Estonia, Finland, Ireland, Italy, Luxembourg, and Portugal). They found that VOC cases needed hospitalization (B.1.1.7, 11.0%; B.1.351, 19.3%; and P.1, 20.0%) and ICU admittance (B.1.1.7, 1.4%; B.1.351, 2.3%; and P.1, 2.1%) more frequently than non-VOC cases (hospitalization for 7.5%, ICU admittance for 0.6%). People hospitalized for the B.1.1.7 variant infection had significantly lower ages (mean age 63 years; median age 65 years) compared to those with non-VOC variants (mean age 69 years, median age 75 years), but also compared to those with other VOC variants, such as B.1.1.351 (mean and median age 67 years) and P.1 (mean age 71 years, median age 76 years). There were no significant differences regarding the risk of death.

The B.1.617.2 variant appeared for the first time in India in December 2020 and spread rapidly in the following months, becoming a new Variant of Concern. This variant is characterized by several mutations in the spike protein, in particular T19R, L452R, T478K, D614G, P681R, and D950N [178]. These mutations can be of particular concern: among all, D614G (a mutation strongly shared with other Variants of Concern) can be responsible for increased transmission, whereas L452R may impair neutralization by antibody response [179,180]; according to a study by Motozono et al. [174], the L452R mutation can also impair HLA-24-restricted cellular immunity, favoring viral infectivity and its replication inside human cells. According to preliminary data given by a study from Bernal et al. [175], vaccines BNT162b2 and ChadOx1 maintain efficacy against the 1.617.2 variant, with only slightly reduced capability in preventing symptomatic disease compared to the B.1.1.7 variant.

### Summary

Prevalence of these variants is increasing all around the world, Italy included. According to the Istituto Superiore di Sanità (ISS, Rome, Italy), between 28 December 2020 and 19 May 2021, genotyping of 23,170 cases of SARS-CoV-2 infection identified 73.06% of cases belonging to the B.1.1.7 variant, 6% belonging to P.1 lineage, 1.17% belonging to the 1.525 variant (Nigerian variant), and less than 1% belonging to other variants under monitoring (such as B.1.351, P.2, B.1.1.7 + E484K, and B.1.617.1/2); 18.9% of cases did not belong to the variants currently monitored [181].

## 10. The Impact of Variants on Monoclonal Antibodies, Convalescent Plasma, and Vaccines

### 10.1. The Impact of Variants on Monoclonal Antibodies and Convalescent Plasma

Monoclonal antibodies represent a recent therapeutic strategy targeting the RBD domain of the SARS-CoV-2 spike protein [182]. Supasa et al. [167] highlighted that the B.1.1.7 variant needed higher neutralizing titers in convalescent plasma or vaccinated sera to be effective. At present, there is no evidence this variant can escape the serum neutralization elicited by previous SARS-CoV-2 infections or available vaccines, although the neutralizing capacity may be less effective due to the N501Y mutation, especially when considering IGHV3-53 monoclonal antibodies. In addition, B.1.1.7 contains other changes, such as 69–70 and 144 deletions in the N-terminal region, which could be important in neutralization [183,184,185].

Zhou et al. [168] demonstrated that the B.1.351 strain resulted more difficult to neutralize when compared to the archaic strain. Of the monoclonal antibodies, most were characterized by a reduced efficacy, up to a complete loss of function for some of them; other antibodies maintained neutralizing activity. Zhou et al. also analyzed the neutralization of convalescent plasma, where they noticed a drop in neutralization titers of 13.3 against the B.1.351 strain; in some cases, convalescent plasma was ineffective. The B.1.351 strain is less sensitive to neutralization when compared to the B.1.1.7 strain, even if they share the N501Y mutation [186]. 

Planas et al. [166] compared sensibility to antibody neutralization of authentic viral variants D614G, B.1.1.7, and B.1.351. They found out that mAb 102 neutralized all strains in a similar way, while mAb 48 neutralized D614G, but it was inactive against B.1.1.7 and B.1.351, confirming that these variants are characterized by reduced sensibility due to mutations that impair binding between antibodies and their target. Planas et al. also considered convalescent plasma neutralizing efficacy against these three viral strains, taking samples 3 and 6 months after SARS-CoV-2 infection [166]. They found similar sensibility against D614G and B.1.1.7 strains; for the B.1.351 strain, they noticed a significative 5- to 10-fold reduction in neutralizing titers compared to D614G and B.1.1.7 strains. Most of the convalescent plasmas could neutralize all three strains 3 months after the original infection; 6 months after infection, the fraction of neutralizing antibodies started to decrease, especially for B.1.351. 

### 10.2. The Impact of Variants on Current Vaccinations

Several efforts are made to develop vaccines to face the SARS-CoV-2 pandemic. These vaccines aim to enhance a humoral- and cell-mediated immune response against the SARS-CoV-2 S protein. They have been developed based on the original strain first identified in Wuhan, and they are based on several mechanisms, including mRNA-based technologies, virally vectored platforms, inactivated adenovirus, and recombinant proteins [168,187]. Currently, four vaccines are licensed by regulatory agencies, and they use RNA or viral vector technologies. According to a study conducted by Muik et al. [187], human sera collected from individuals vaccinated with Pfizer-BioNTech Comirnaty can neutralize pseudovirus of SARS-CoV-2 lineage B.1.1.7 with neutralizing titers slightly reduced, even if mostly preserved, when compared to the Wuhan strain. Xie et al. [186] demonstrated that some mutations of the B.1.1.7 variant had a low effect on neutralization produced by the sera of people vaccinated with two doses of the Pfizer vaccine, even if this study was not conducted using all mutations of the B.1.1.7 variant. According to Collier et al. [187], sera from people vaccinated with Pfizer did not show significant reduced inhibition of the parental pseudovirus or pseudovirus carrying three mutations in the S protein, including H69/V70del, N501Y, and A570D. Neutralizing titers were reduced against the pseudovirus carrying all mutations of B.1.1.7. In this setting, no loss of neutralizing activity against B.1.1.7 by sera from people previously vaccinated with Moderna or Pfizer was demonstrated by Wang et al. [188], and Wu et al. [176] did not find a significant impact on neutralizing capability of individuals or primates vaccinated with the mRNA-1273 vaccine (Moderna vaccine) against the B.1.1.7 variant. Overall, these studies suggest that the efficacy of administrated vaccines is similar or slightly reduced against the B.1.1.7 variant compared to the original strain, even if there are study limitations; in particular, these studies only consider the humoral immune response, and other studies need to be conducted [158]. Zhou et al. [168] demonstrated reduced neutralizing titers against the B.1.351 variant developed after AstraZeneca and Pfizer vaccination, respectively, a 9-fold and 7.6-fold reduction. Data from Zhou’s study suggest that efficacy is partly preserved, in particular for the Pfizer-BioNTech vaccine. Recent data suggest that the Novavax vaccine, characterized by 95.6% efficacy against the ancestral SARS-CoV-2 strain and 85.6% efficacy against the B.1.1.7 variant, reaches only 60% activity in South Africa, where 92.6% of infections are sustained by the B.1.351 variant. The Janssen monodose vaccine, which prevents moderate and severe disease in 72% of cases, preserved only 57% of its activity in South Africa. According to a report by Madhi et al. [173], the AstraZeneca vaccine only reaches 10.4% efficacy in preventing moderate and severe disease in B.1.351 variant infections. The Moderna mRNA-1273 vaccine induces antibody production against other SARS-CoV-2 variants, although its efficacy is reduced 5-fold to 10-fold against the S protein of B.1.351 lineage compared to a pseudovirus carrying the D614G mutation [176]. According to a study by Planas et al. [166], sera collected from people vaccinated with Pfizer Comirnaty neutralized the D614G-mutated strain at week 2 after the first dose of vaccination; B.1.1.7 began to be neutralized three weeks after the first dose of vaccination, even if less effectively compared to the D614G-mutated strain. D614G and B.1.1.7 strains were similarly neutralized after 4 weeks (1 week after second dose of vaccination). The immune response against B.1.351 became detectable at week 4, but neutralizing titers were 14-fold and 53-fold lower when compared, respectively, to D614G and B.1.1.7 strains.

A recent study conducted by Bernal et al. [175] compared the effectiveness of BNT162b2 (Pfizer-BioNTech Comirnaty vaccine) and ChAdOx1 (AstraZeneca vaccine) COVID-19 vaccines against the B.1.617.2 variant and the B.1.1.7 variant. They found a slight reduction in vaccine efficacy preventing symptomatic disease in the B.1.617.2 variant compared to B.1.1.7; in particular, this difference was very small after the administration of two doses of the vaccine. Overall, the effectiveness of two doses of the ChAdOx1 vaccine was lower than that of two doses of BNT162b2 against both variants.

## 11. Vaccine-Associated Adverse Events

To date, four vaccines have been authorized by international agencies, such as the European Medicines Agency (https://www.ema.europa.eu/en—EMA, accessed 12 August 2021). Two are DNA vaccines, the AstraZeneca (now Vaxzevria) vaccine and the Janssen vaccine, which are administered in two doses or one dose, respectively (the Oxford–AstraZeneca ChAdOx1 vaccine produced by the British University of Oxford, British–Swedish company AstraZeneca, and the Coalition for Epidemic Preparedness Innovations consisting of a replication-deficient chimpanzee adenoviral vector and the Janssen Ad26.COV2.S vaccine produced by Janssen Pharmaceutical (a subsidiary of Johnson & Johnson) and the Beth Israel Deaconess Medical Center composed of a recombinant vector based on human adenovirus type 26 incompetent for replication, suitably modified to contain the gene encoding for the complete sequence of the SARS-CoV-2 S protein in a stabilized conformation); the other vaccines are two mRNA vaccines (the Pfizer–BioNTech vaccine also known as Comirnaty (BNT162b2) produced by the German company BioNTech and the American company Pfizer and the Moderna Spikevax COVID-19 vaccine produced by the American company Moderna, the U.S. National Institute of Allergy and Infectious Diseases, the U.S. Biomedical Advanced Research and Development Authority, and the Coalition for Epidemic Preparedness Innovations). The mRNA vaccines are administered in two doses separated from each other by an interval of 3 weeks.

Other vaccines less used for vaccination purposes are two vector vaccines (Sputnik V from Gamaleya Research Institute, Ad5-nCoV from CanSino), four conventional inactivated vaccines (BBIBP-CorV from Sinopharm, BBV152 from Bharat Biotech, CoronaVac from Sinovac, and CoviVac), and two protein subunit vaccines (EpiVacCorona and ZF2001 from Vektor Institute). All currently authorized and recommended COVID-19 vaccines are safe, effective, and reduce the risk of severe illness. In fact, their administration has been associated with a strong decrease in SARS-CoV-2 infections and associated deaths [189,190]. However, in parallel with these results, some adverse events have also been described. 

The Center for Disease Control and Prevention (CDC) quickly adapted its proven monitoring systems by creating a new post-authorization safety surveillance tool, V-safe, designed and deployed specifically to acutely track vaccinated individuals, including populations of pregnant women who were also surveyed through a personalized, real-time registry (https://www.cdc.gov/coronavirus/2019-ncov/vaccines/safety/vsafe.html, accessed 12 August 2021). Similarly, the EMA and the Italian Medicine Agency (https://www.aifa.gov.it/content/segnalazioni-reazioni-avverse-AIFA, accessed 12 August 2021) also represent important adverse reaction monitoring systems.

In general, most of the reported side effects of either DNA or mRNA COVID-19 vaccines are mild or moderate and short lived [191,192]. These include fever, fatigue, headaches, muscle aches and pains, chills, vomiting or diarrhea, flu-like symptoms, localized soreness in the area of the injection, redness at the site of inoculation (the arm), and enlargement of ipsilateral axillary lymph nodes. They usually disappear within 2 to 3 days with the only exception of lymph node enlargement, which may persist for a few more days. The likelihood of occurrence of each of these side effects varies by vaccine (https://www.cdc.gov/coronavirus/2019-ncov/vaccines/safety/vsafe.html, accessed 12 August 2021). The two mRNA vaccines were the first to be administered, and for these vaccines as well as for DNA vaccines, some cases of hypersensitivity adverse reactions have been reported [193,194]. Both Comirnaty and Spikevax vaccines are administered to children and adults 12 years of age or older and have shown excellent safety and efficacy in phase 3 trials [193]. With the Comirnaty vaccine, rare severe effects (one case per 1000 people) may be temporary paresis of one side of the face or allergic reactions, such as hives or facial swelling. In very rare circumstances and especially in young males vaccinated with the same vaccine, cases of myocarditis and pericarditis in the 2 weeks following vaccination may occur [195]. More serious or long-lasting side effects may also occur with extreme rarity. The occurrence of these rare events is continuously monitored for all vaccines by all medicine agencies.

In addition to minor adverse effects described for all vaccines authorized to date, events of thrombosis, thrombocytopenia, and hemorrhage have also been described in close temporal proximity to the administration of Vaxzevria and Janssen vaccines, although cases of thrombotic events have also been described in close temporal proximity with the administration of mRNA vaccines [191,194,196]. They have been emphasized in the media but are actually very rare adverse events (7 cases per 1 million among vaccinated women aged 18–49 years). In these cases, thrombosis has been found, usually at unusual sites, especially at the cerebral or abdominal venous level, accompanied by thrombocytopenia and the appearance of a particular type of autoantibody (anti-PF4 antibodies), which are partly responsible for platelet activation and reduction in platelet number [192,197]. In this regard, vaccine-induced immune thrombocytopenia (VITT), a serious adverse effect observed after the administration of the Vaxzevria vaccine and the Janssen COVID-19 vaccine associated with unusual thrombosis, has been described. VITT is caused by anti-PF4 antibodies that activate platelets through their FcgRIIa receptors [198].

For these reasons, the Vaxzevria vaccine is only administered to individuals over the age of 60 after written consent. The Janssen vaccine is administered to individuals 18 years of age or older in one dose only, and, similar to the Vaxzevria vaccine, it is strongly recommended to people over 60 years of age. Following vaccination with the Janssen vaccine, alongside the well-known short-term adverse effects that can occur as with other vaccines, very rare cases of capillary leak syndrome (CLS) have been reported. This is a very serious clinical condition that causes fluid to leak from the capillaries resulting in rapid swelling of the arms and legs, sudden weight gain, and hypotension, which can lead to death (https://www.cdc.gov/coronavirus/2019-ncov/vaccines/safety/vsafe.html; https://www.aifa.gov.it/content/segnalazioni-reazioni-avverse-AIFA, accessed 12 August 2021).

Other rare and undesirable effects described with the Janssen vaccine or Vaxzevria may be urticaria; severe allergic rash; and the appearance of clots at unusual sites, such as the brain, intestine, liver, and spleen, accompanied by thrombocytopenia. However, the benefit of the DNA vector and mRNA vaccines in preventing severe SARS-CoV-2 continues to outweigh the risk of side effects. From the data currently available, all vaccines used in Europe and the United States (Pfizer/BioNTech, Moderna, Vaxzevria/AstraZeneca, and Janssen/Johnson & Johnson) are effective against variants. With regard to the Delta variant, however, protection is greater with the full vaccine cycle: those who have received only the first dose of a vaccination involving two administrations are less protected against infection of the Delta variant than against infection of other variants, regardless of the type of vaccine administered (https://www.humanitas.it, accessed 12 August 2021; https://www.epicentro.iss.it/coronavirus/sars-cov-2-monitoraggio-varianti, accessed 12 August 2021). On the other hand, completing the vaccination cycle offers protection against the Delta variant almost equivalent to that observed against the Alpha variant.

### 11.1. Antibody-Resistant SARS-CoV-2 Variants

It has been recently reported that most of the of the SARS-CoV-2 genome is not under positive selection [199], as this virus is commonly considered to acquire mutations more slowly than other RNA viruses. However, since neutralizing antibodies (nAbs) against SARS-CoV-2 are broadly present in humans soon after the beginning of the pandemic, single point mutations can provide resistance to nAbs. Therefore, SARS-CoV-2 can potentially expand rapidly under positive selection pressures as it might develop mutations as an immune evasion strategy. Recent evidence indicates that single point mutations within the RBD can led to resistance [159] to neutralizing convalescent plasma from multiple donors [200]. Indeed, RBD is the key region for nAbs binding. This is understandable, as nAb binding to this region can prevent the interaction between the S protein and the ACE2, thereby impairing viral infection. This may suggest that specific single SARS-CoV-2 mutants may potentially be able to evade S-targeting vaccinal immunity, ultimately leading to the propagation of vaccine-resistant SARS-CoV-2 variants. 

Mutations at position 477 of the spike protein, i.e., S477G, S477N, and S477R, have been reported to rank prominently among nAb escape mutations identified [201,202]. Additionally, S477G also conferred resistance to several tested sera [201,202]. However, it is important to point out that mutations at position 477 have not been identified as being important with convalescent plasma [203]. A recent report also identified E484 and S494 mutations, which are capable of interacting with nAbs but not with ACE2, as determinant for promptly evolved immune escape mutants [204]. In addition, it has also been reported that the combination of these mutations with others promoting ACE2 binding, such as N501Y, increases their ability to escape nAb responses. The mutation at position 494 also deserves attention, either alone or combined with synergetic mutations, as it seems to reduce the neutralization competency of convalescent sera, thereby facilitating nAb escape, and thus facilitating antibody escape without modifying the affinity for ACE2 [204].

### 11.2. Summary

The development and distribution of vaccines against SARS-CoV-2 should always consider the question of mutational escape from antibody prophylaxis to the forefront. With the emergence of the identification of newly emerged SARS-CoV-2 variants, which might potentially harbor altered transmissibility and immune evasion potential, researchers should always evaluate the SARS-CoV-2 escape potential to nAbs targeting the S protein [205]. The potential rapid evolutionary evasion of nAbs by SARS-CoV-2 should force the continuous development of biomedical interventions aimed at bringing the virus under control, namely the risk of reduced vaccine efficacy over time as vaccine-resistant variants continue to emerge.

## 12. Concluding Remarks and Future Perspectives

The above studies have improved our knowledge behind the identification of the natural reservoir, which acts as an intermediate animal host, as well as presenting important implications for understanding zoonotic transmission of SARS-CoV-2 and both human-to-human and human-to-animal transmission. Further studies will be needed to evaluate the real efficacy of vaccination against SARS-CoV-2 and its new variants that could potentially be able to escape the humoral immune response making the vaccination campaign ineffective. In this context, current vaccines will have to be updated soon, because taking into account that it will take a long time to vaccinate the entire general population, including young people, adults, and elderly individuals, SARS-CoV-2 could quickly lead to the appearance of new mutations that could negatively impact existing vaccines. Currently, more than 200 vaccines are being tested worldwide. However, to counter the current pandemic, it was essential to immediately administer the vaccines produced to date, which, from the current data, although fragmentary, would seem to be able to neutralize all the variants that have emerged to date, including the South African and Indian one. According to WHO, SARS-CoV-2 has infected nearly 173 million people and killed over 3.72 million since April 2020 (https://www.who.int/emergencies/diseases/novel-coronavirus-2019/situation-reports, accessed 12 August 2021). COVID-19 is an immense threat to global public health, and as long as the virus is circulating, we cannot consider ourselves safe. Vaccines are the most cost-effective and effective strategies to control and prevent the infection of most infectious diseases, and COVID-19 is among them.

## Figures and Tables

**Table 1:** Function of 16 non-structural proteins (NSPs) translated from the CoV genome. NSP1-10 is translated from ORF1A, whereas NSP11-16 is translated from ORF1B.

Gene	Non-Structural Protein (NSP)	Function
ORF 1A	NSP 1	Inhibits interferon signaling Inhibits host protein synthesis Cellular mRNA degradation
	NSP 2	Unknown
	NSP 3	Tethers genome to RTC allowing initiation of RNA synthesis Papain-like protease (PLP) for polypeptide cleaving Inhibit host innate immune response Promotes cytokine expression
	NSP 4	Transmembrane helices that anchor RTC to intracellular membranes Double membrane vesicle formation
	NSP 5	Main protease (Mpro) for polypeptide cleaving Chymotrypsin-like protease (3CLpro) for polypeptide cleaving Inhibits interferon signaling
	NSP 6	Transmembrane helices that anchor RTC to intracellular membranes Double membrane vesicle formation
	NSP 7	Essential small proteins: Hexadecameric complex (cofactor NSP8 and NSP12)
	NSP 8	Essential small proteins:Essential small proteins: Hexadecameric complex (cofactor NSP7 and NSP12) Primase
	NSP 9	Essential small proteins: RNA-binding protein Dimerization
	NSP 10	Essential small proteins: Zinc-binding domain (ZBD) cofactor for 2′-O-methyltranferase (2′-O-MTase) Scaffold protein for NSP14 and NSP16
ORF 1B	NSP 11	Unknown
	NSP 12	RNA-dependent polymerase (RdRP)
	NSP 13	Zinc-binding domain (ZBD) RNA 5′ triphosphate—synthesis of 5′ terminal cap structure of mRNA RNA helicase—unwinds RNA duplexes with a 5′-3′ polarity
	NSP 14	3′-5′ exonuclease—some proof-reading activity that is unique to CoV N7-methyltransferase
	NSP 15	Endoribonuclease

**Table 2:** Variants of Concern according to the latest classification by ECDC [165].

Variants of Concern		
Lineage and Addition Mutation	Country First Detected	Spike Mutation of Interest
B.1.1.7	United Kingdom	N501Y, D614G, P681H
B.1.1.7 + E484K	United Kingdom	E484K, N501Y, D614G, P681H
B.1.351	South Africa	K417N, E484K, N501Y, D614G, A701V
P.1	Brazil	K417T, E484K, N501Y, D614G, H655Y
B.1617.2	India	L452R, T478K, D614G, P681R

**Table 3:** Variants of Interest according to the latest classification by ECDC [165].

Variants of Interest		
Lineage and Addition Mutation	Country First Detected	Spike Mutation of Interest
B.1.525	Nigeria	E484K, D614G, Q677H
B.1.427/B.1.429	USA	L452R, D614G
P.3	The Philippines	E484K, N501Y, D614G, P681H
B.1.616	France	V483A, D614G, H655Y, G669S
B.1.617.1	India	L452R, E484Q, D614G, P681R
B.1.617.3	India	L452R, E484Q, D614G, P681R
B.1.620	Unclear (b)	S477N, E484K, D614G, P681H
B.1.621	Colombia	R346K, E484K, N501Y, D614G, P681H

## Data Availability

Data sharing is not applicable to this article as no new data were created or analyzed in this study.
